# Compound climate extremes and “once-in-a-century” risk communication

**DOI:** 10.1016/j.lanwpc.2026.101973

**Published:** 2026-09-01

**Authors:** John S. Ji, Qihong Deng

**Affiliations:** a Vanke School of Public Health, Tsinghua University, Beijing, China; b School of Environment, Tsinghua University, Beijing, China; c School of Architecture and Urban Planning, Zhengzhou University, Zhengzhou, China; d School of Public Health, Zhengzhou University, Zhengzhou, China

In July 2026, China's national meteorological and water resources authorities issued red alerts for flash floods and geological hazards across Liaoning, Jilin, and Anhui provinces, the ceiling of the country's four-level warning system, as the remnant circulation of Typhoon Bavi pushed torrential rain into the northeast, a region not historically associated with typhoon-driven flooding of this scale.^1^, ^2^, ^3^ Days earlier, Tropical Storm Maysak had triggered reservoir breaches and large-scale evacuations more than 1500 km to the south, in Guangxi.^4^ Official sources described the month's disaster risk as “complex,” with typhoons, rainstorms, floods, geological hazards, heatwaves, and drought now overlapping within the same weeks, sometimes the same days, rather than taking polite turns on the seasonal calendar.^5^ Even as the northeast raised flood emergency responses to their highest level, Xinjiang, in the arid northwest, had already recorded temperatures near 50 °C in June.^5^

Meteorological strain wreck. So much for “once-in-a-century.”

This is no longer about any single storm. It is a new normal: extreme events once described as “once-in-a-century” are increasingly compound, concurrent, and geographically dispersed, arriving faster than the institutions and infrastructure built to manage them.

The term “once-in-a-century” is shorthand for an event with a 1% chance of occurring in any given year. Hydrologists have long recognised that this language is easily misinterpreted: it describes a statistical probability, not a promise that such an event will occur only once every hundred years.^6^^,^^7^ Climate change has rendered the historical baseline obsolete. On 20 July 2021, Zhengzhou recorded 201.9 mm of rainfall in a single hour, a new national record, with three-day cumulative rainfall of 617.1 mm, approaching the city's entire annual mean.^8^ The event overwhelmed a city built, in part, under China's “sponge city” programme, designed explicitly to absorb such extremes.^8^ Four years later, in August 2025, Zhengzhou's flood-control emergency response was again raised to Level III.^9^ The hundred-year flood had taken four years to make its second appearance.

These events carry direct health consequences–drowning, injury, and disrupted access to routine and emergency care–and globally, public health systems have developed real capacity to respond to the acute aftermath of a single flood. For instance, China's Centres for Disease Control and Prevention issues detailed post-flood guidance on indoor mould identification and remediation, with explicit precautions for vulnerable groups: pregnant women, children under 12, adults over 65, and people with asthma.^10^ This is well-designed, evidence-based guidance. But it is guidance calibrated to a single, bounded event: dry the building, remove saturated porous materials within 48 h, ventilate, protect vulnerable residents during cleanup. It says nothing about a household, or a health system, absorbing a second or third such event within years rather than decades, nor about heat and flood advisories issued in the same week to the same population. Adaptation guidance built for isolated disasters is necessary but insufficient for compound, recurring ones.

Three shifts follow from this pattern. First, risk communication should retire probabilistic century-scale language in public-facing materials in favour of terms that convey trend and recurrence, such as “increasingly frequent severe rainfall” rather than “once-in-a-century flood,” so that recurrence within a decade is not received as a statistical anomaly requiring no institutional response. Second, infrastructure and early-warning design standards, including within “sponge city” and reservoir safety programmes, should incorporate non-stationary risk models that use recent-period distributions, rather than full-record historical baselines that dilute the recent shift toward higher-intensity rainfall. Third, public health guidance should evolve from single-event response protocols toward compound-event and cumulative-exposure frameworks, addressing populations facing sequential or simultaneous heat and flood risks within the same season, as well as the mental and physical health burdens of repeated displacement and recovery.

Experts and meteorological authorities have officially acknowledged a warming trend that outpaces the global average and a rise in the frequency of extreme events.^5^ Public health guidance and infrastructure standards, however, continue to speak as if these events remain rare. This summer made the mismatch impossible to ignore. Flood warnings in the northeast and heat alerts in the northwest arrived in the same week. The climate has changed. The language needs to.

## Declaration of interests

None.
